# SUPPORTING SOCIAL INTERACTION OF OLDER FAMILY CARE PARTNERS THROUGH A DIGITAL HEALTH PHYSICAL ACTIVITY PROGRAM

**DOI:** 10.1093/geroni/igae098.3384

**Published:** 2024-12-31

**Authors:** Dawon Baik

**Affiliations:** University of Colorado, Aurora, Colorado, United States

## Abstract

Older Family care partners (FCPs) experience more social isolation, mental and physical health issues than younger FCPs. Loneliness and social disconnectedness in older FCPs can lead to an increased risk of poor health and low well-being. To support social interaction and connectedness in older FCPs of persons with heart failure (HF-FCPs), we identified their needs and preferences on a technology-supported physical activity (PA) program using scenario-based design methods. The program included one-on-one PA coaching, using a fitness tracker and motivational text messaging. Fifteen HF-FCPs were recruited at a University Hospital between September and December 2022. We conducted semi-structured qualitative interviews. Participants were aged 65.9±4.4 years (M±SD), female (73%), and spouses (87%). HF-FCPs perceived that the program could support their social connectedness. Participants reported that interaction with a health coach via videoconferencing would be valuable for their social health in the context of caregiving (e, g., time constraints, caregiving duties limiting FCPs to the home). A group-based program was suggested to increase motivation for support and feedback from other FCPs. They indicated that texting would be useful to share their PA and caregiving experience and provide support and encouragement to other FCPs. Participants highlighted the need for their own social groups, because as one described: “There is a support group for people with the same condition and their caregivers, but it’s really talking about the condition itself, not about how we take care of ourselves.” Future studies should develop and test technology-based, caregiver-centered interventions for sedentary and socially isolated older FCPs.

